# Center or periphery? Modeling the effects of focal adhesion placement during cell spreading

**DOI:** 10.1371/journal.pone.0171430

**Published:** 2017-02-03

**Authors:** Magdalena A. Stolarska, Aravind R. Rammohan

**Affiliations:** 1 Department of Mathematics, University of St. Thomas, St. Paul, MN, United States of America; 2 Advanced Modeling and Analysis, Corning Incorporated, Corning, NY, United States of America; Seoul National University College of Pharmacy, Republic of Korea

## Abstract

Focal adhesions are often observed at the cell’s periphery. We provide an explanation for this observation using a system-level mathematical model of a cell interacting with a two-dimensional substrate. The model describes the biological cell as a hypoelastic continuum material whose behavior is coupled to a deformable, linear elastic substrate via focal adhesions that are represented by collections of linear elastic attachments between the cell and the substrate. The evolution of the focal adhesions is coupled to local intracellular stresses which arise from mechanical cell-substrate interactions. Using this model we show that the cell has at least three mechanisms through which it can control its intracellular stresses: focal adhesion position, size, and attachment strength. We also propose that one reason why focal adhesions are typically located on the cell periphery instead of its center is because peripheral focal adhesions allow the cell to be more sensitive to changes in the microenvironment. This increased sensitivity is caused by the fact that peripherally located focal adhesions allow the cells to modulate its intracellular properties over a much larger portion of the cell area.

## Introduction

Cell based assays are increasingly becoming an important part of drug development where biological cells are placed in either functionalized petri dishes or microplates of different formats, for example 96 well plates [1, 2]. The key to the success of these cell based assays is that the functionalized surfaces allow the cells to behave as similarly as possible to their native *in vivo* environments. Cells which behave most naturally can then be used to assess the performance of candidate drug molecules in their ability to activate or deactivate certain biological pathways.

Effective design of these functionalized surfaces requires a fundamental understanding of the interaction between a cell and the surface. Adherent cells engage with the underlying substrates (the extracelluar matrix—ECM—*in vivo*, or a functionalized surface with peptide coatings *in vitro*) through the formation of focal adhesions (FAs). FA formation involves sensing the chemical and mechanical microenvironment on the substrate by cell surface receptors, which for many cell types are integrins, that bind to specific chemical motifs in the ECM/functionalized surface. Once this binding occurs, a complex orchestration of many different molecules occurs intracellularly, which results in the accumulation of over seventy proteins at this cell-surface junction [3, 4]. These protein complexes are referred to as focal adhesions, which are both chemically and mechanically sensitive. The mechanical sensitivity is reflected in detailed experimental studies which show that the size of the focal adhesion complexes grow with the stiffness of the underlying substrates [5]. The chemical sensitivity has also been well established with different chemical motifs patterned on the surfaces [6].

In the last decade FAs have been characterized extensively from an experimental perspective [7, 8]. Of the different aspects of FAs, such as their growth, dynamics, and mechanosensitivity, one which is often observed but is not well understood is the location of FAs on the contact region between the cell and substrate. When cells are imaged on flat surfaces it is almost always observed that the FAs are localized at the cell periphery and rarely seen in the cell center [9–12]. Recent experimental works have begun to elucidate the effects of FA positioning on cell behavior. Elineni et al. [10] have shown that the attachment strength of cells increases when FAs are found in the periphery versus in the center. Other studies show that increases in cell spread area, FA size, and FA location collectively lead to an increase in cell traction stresses [13]. These results raise the question about how FA position affects intracellular mechanics and the cellular response to its environment.

Existing models look at certain specific aspects of FAs. For example, one-dimensional models of only the FA region aim to understand how FA sizes and positions respond to forces [14, 15]. Models by Olberding et al. [16] and Walcott et al. [17] use a thermodynamic approach to capture the active and inactive state of the integrin and link integrin activation to the growth of FAs but do so using a very simplified representation of the cell. One dimensional models of single cell motility have elucidated the bell-curve shaped relationship between adhesive strength and cell velocity [18]. Others have developed two dimensional models of cells and have incorporated FAs into the models. However, the aim of these models is to better understand the evolution of stress fibers, contractile actin bundles [19, 20], the complex interplay of various cellular components required for cell motility [21, 22], or the effects of FA maturity on spontaneous cell migration [23]. However, none of these works focus on the effects of growth and location of FAs on cell movements.

Given these existing models, in this paper we set out to describe a two-dimensional representation of a spreading cell as a mechanical body with the detailed biochemistry associated with the FA’s chemical and mechanical response captured by a mathematical model that has been simplified so that all key physical aspects of cell-substrate attachments are captured. The effects of the actin polymerization and actomyosin contraction, which serve as the primary motile machinery of the cell and are controlled by a host of accessory proteins [24], are also modeled in an approximate way by specifying the cell’s rate of active deformation. These simplified descriptions of FA evolution and cytoskeletal deformation, in conjunction with the two dimensional mechanical description of the cell, allow us to develop a deterministic system level model of the cell. Further in this model description we are also able to explicitly account for the deformation of the substrate and model its mechanical response to the forces exerted on it by the cell. This allows us to compare the model predicted substrate deformations with experimentally determined traction force microscopy measurements, which allows us to calibrate and validate our simulations. Therefore, our aim in this paper is two-fold. First, we wish to illustrate that our model of the cell-substrate system can be used for a variety of cell types spreading over different environments. Second, we use this description to investigate the effect of having FAs at the cell periphery versus its center. Specifically we investigate the effect of FA size, location, strength of attachment, and substrate stiffness on cellular and substrate response, which is a step towards understanding why FAs are more often found on a cell’s periphery rather than its center.

## Methods

**Fig 1:**
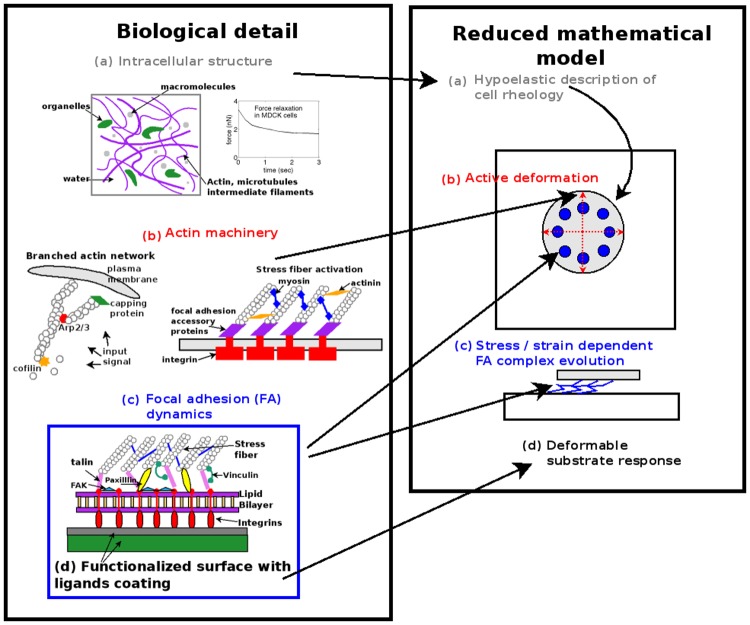
Cell-substrate interaction model is illustrated on the right. The biological details of the mechanisms involved in cell spreading are reduced to four major model components, labeled (a)-(d) in the right panel titled “Reduced mathematical model”. Both the cell and the substrate are two dimensional. The rheology of the cell is determined by local concentrations of filaments, organelles, and additional macromolecules, and upon applying an external force via microindentation, the cytoplasmic forces undergo relaxation [26]. We assume the average material properties of the cell to be hypoelastic (a), and we assume that the deformable substrate is linearly elastic (d). Actin-based cell movement [24, 27] is described by an active rate of deformation tensor (b). The interaction between cell and substrate occurs via the FAs, which are modeled as collections of discrete linear springs (c). A description of the dynamic FA complex is simplified and written as stress dependent evolution of a single scalar field representing the volume fraction of the entire FA complex [4].

The model of the cell-substrate system consists of four components, as illustrated in Fig 1. To model the averaged response of the mixture containing cytoskeletal filaments, organelles, cytosol, and other intracellular macromolecules we describe the cell as a hypoelastic material [25] (component (a) in Fig 1). The reorganization of the actin cytoskeleton to drive cell movement and generate intracellular stresses is captured in the model by a single rate of deformation tensor field which describes the active local deformations of the cell (component (b)). The cellular FAs and the forces therein are incorporated into the model as a collection of discrete linear springs which attach the hypoelastic cell to the substrate (component (c)). We assume the mechanical response of the substrate to be linearly elastic since its deformations are generally small (component (d)). The model describing the evolution of FAs and all its accessory proteins is written so that the position of FAs is described by a single, stress-dependent scalar. Our aim in this work is to include key qualitative and quantitative elements of cell movement, cell adhesion, substrate response, and the interaction between these processes in a relatively simple manner, but one that reflects the essential physical interactions required to simulate experimentally observable behavior.

### Cell mechanical model

We treat the cell as a two-dimensional, deformable continua. The material response of the cell is assumed to be governed by a hypoelastic model, which is equivalent to linear elasticity for small strains, while for large strains accounts for flow-like inelastic effects of large deformation. Hypoelastic constitutive equations have been shown to be thermodynamically consistent [28] and valid for biological materials [29, 30] due to the fact that biological systems involve growth, material remodeling, and large deformations [31]. The finite element analysis of these features, pursued in this paper, benefits from occasional remeshing (described later), which requires associated transfer of the fields involved between the computational meshes. This remeshing process also becomes simpler in the context of hypoelastic materials.

The equilibrium and constitutive equations for the cell are, respectively, given by

$$\nabla \cdot \sigma_{c}\;-\;k_{a}\left(u_{c}-u_{a}-u_{s}\right)\delta \left(x-x_{c}\right)\;=\;0$$ (1)

$${\overset{\nabla }{\sigma }}_{c}=C_{c}\left(\frac{1}{2}\left(\nabla v_{c}+\nabla^{T}v_{c}\right)-D^{A}\right),$$ (2)

where

$${\overset{\nabla }{\sigma }}_{c}\;=\;\frac{d\sigma_{c}}{dt}-\left(\nabla v_{c}\right)\sigma_{c}-\sigma_{c}\left(\nabla^{T}v_{c}\right).$$ (3)

The Cauchy stress tensor within the cell is ***σ***_*c*_, and ${\overset{\nabla }{\sigma }}_{c}$ is the Oldroyd time derivative to render the constitutive equation frame-invariant. Therefore, Eq (2) combined with Eq (3) describe the material response of the cell, in which the active rate of deformation is denoted by the tensor field **D**^*A*^. The active rate of deformation is discussed in the next section. The second term on the left of Eq (1) represents the force from the FA spring acting on the cell. The displacements of the cell and the substrate, respectively, are **u**_*c*_ and **u**_*s*_, and the velocity of the cell is represented by $v_{c}=\frac{du_{c}}{dt}$. The cell displacement at the time a FA spring binds to the substrate is represented by **u**_*a*_. Since the displacements of the substrate are small relative to those of the cell, we do not explicitly account for its displacement at the moment of attachment in the model equations. The FA spring stiffness is *k*_*a*_, which we set to 5 × 10^−5^ dynes/*μ*m [32]. The location at which the FA spring is connected to the cell is given by **x**_*c*_, and *δ*(⋅) is the Dirac delta function. The cell boundary is traction free.

The constant Hooke tensor representing the material properties of the cell is given by **C**_*c*_. Since the cell thickness in the direction normal to the substrate is small compared to its radius, so we assume that the cell is in a state of plane stress, which for isotropic materials assumed here, leads to the Hooke tensor that has the following matrix representation in any Cartesian coordinate system

$$C_{c}=\frac{E_{c}}{1-\nu_{c}^{2}}\;\left[\begin{array}{ccc} 1 & \nu_{c} & 0\\ \nu_{c} & 1 & 0\\ 0 & 0 & 1-\nu_{c} \end{array}\right]$$ (4)

where *E*_*c*_ and *ν*_*c*_ are the Young’s modulus and Poisson ratio of the cell, respectively.

### Active deformation

In Eq (2), **D**^*A*^ is the active rate of deformation tensor, which characterizes a cell’s local active rate of deformation due to spreading and contraction and needs to be specified. We assume that the the total rate of deformation tensor, **D**, can be additively decomposed into a stress-related passive part, **D**^*P*^, and an active part, **D**^*A*^, such that

$$D=\frac{1}{2}\left(\nabla v_{c}+\nabla^{T}v_{c}\right)=D^{P}+D^{A}$$ (5)

**D**^*A*^ can in general depend on the variables in the model, such as local stress or the concentration of an intracellular biochemical component. Such an additive decomposition is coupled to the assumption that the active deformation component **D**^*A*^ describes only the local unconstrained rate of active remodeling which is stress free, and hypoelastic stress rates in the cell are related only to the passive component, **D**^*P*^. In Eq (2), **D**^*P*^ is written as **D** − **D**^*A*^. Decompositions similar to this were originally used in modeling of small deformations due to thermal expansion in materials and have been used in various models involving growth of biological tissues (see for example [33]).

Biologically, a cell’s motile machinery is dependent on the dynamic structure of the actin network. The filamentous actin at the cell periphery consists of a branched actin network that pushes out the cell’s membrane via polymerization, while the actin network of the interior of spread cells consists of bundled contractile stress fibers that provide tension and structure to the cell [34, 35]. Incorporating these variations in actin structure would result in a spatially and temporally inhomogeneous active rate of deformation. As a first step, in this paper we assume that the cell actively spreads or contracts at a constant, spatially uniform, and isotropic rate, and thus we take **D**^*A*^ to be

$$D^{A}=\left[\begin{array}{cc} \alpha & 0\\ 0 & \alpha \end{array}\right]$$ (6)

We set the value *α* = 0.00725 min^−1^ for spreading. This value is based on Wakatsuki et al. [36] and is chosen so that the diameter of a circular cell approximately doubles over the course of two hours. We estimate the contraction rate to be *α* = −0.001 min^−1^ in order to obtain experimentally observed cell shapes. We assume that the cellular material that is required to allow the cell to spread comes from the cellular regions which are outside of the two-dimensional plane we consider in our simulations.

### Deformable substrate mechanics

The deformation of the substrate is governed by

$$\nabla \cdot \left(\frac{C_{s}}{2}\left(\nabla u_{s}+\nabla^{T}u_{s}\right)\right)+k_{a}\left(u_{c}-u_{a}-u_{s}\right)\delta \left(x-x_{s}\right)\;=\;0$$ (7)

Here, **C**_*s*_ is the Hooke tensor for the substrate, and with suitable choice of values for the Young’s modulus and Poisson ratio, it has the same form as in Eq (4). The location of the FA spring on the substrate is given by **x**_*s*_.

The system Eqs (1), (2) and (7) is coupled through the FA springs that exert forces on both the cell and the substrate, as seen in Eqs (1) and (7). We set the displacement at the edges of the substrate to zero.

### Spatiotemporal evolution of focal adhesions

The model for cellular and substrate deformation described above can be coupled to a model of a full signal transduction network describing many individual intracellular biochemical processes. Here, we employ a simplified approach in which we model the entire FA complex, consisting of the cell-substrate binding proteins, such as integrins, and other accessory proteins, as a single scalar field, denoted by *ϕ*(**x**, *t*). This scalar value is defined at every point on the cellular computational domain, and its local value represents the volume fraction of the FA complex.

The model for the evolution of the FAs employed here is based on earlier work by Besser and Safran [15]. We note that we choose this model as a basis for our FA evolution in part because of its simplicity. In our future work we plan on including a more detailed description of the FA components. In their one-dimensional model, Besser and Safran represent the entire FA complex by a single scalar-valued function of space and time, and they assume that mechanosensors control the evolution of FA volume fraction by changing the complex’s chemical potential. In their model, the chemical potential depends on changes in conformational energy due to addition of complex units, changes strain energy in the FA complex due to compression and tension within, or on energy change due to molecular deformation. They build their model assuming that compressive stresses in the cell’s cytoskeleton lead to FA activation while tensile stress deactivates the FA. Accordingly, the function *ϕ* is constructed so that compressive stresses increase *ϕ*, and larger values of *ϕ* imply FA activation. Besser and Safran describe the evolution of *ϕ* using

$$\frac{\partial \phi }{\partial t}=C_{1}\left[\mu_{b}-\frac{\epsilon_{b}}{2}\left(\tanh\left(\frac{\beta }{2}\Delta G-\frac{\beta }{2}f_{a}\right)-1\right)-\gamma \left(f_{a}\right)\frac{\partial \phi }{\partial x}+\epsilon \phi -c\phi^{3}+B\frac{\partial^{2}\phi }{\partial x^{2}}\right]$$ (8)

**Fig 2:**
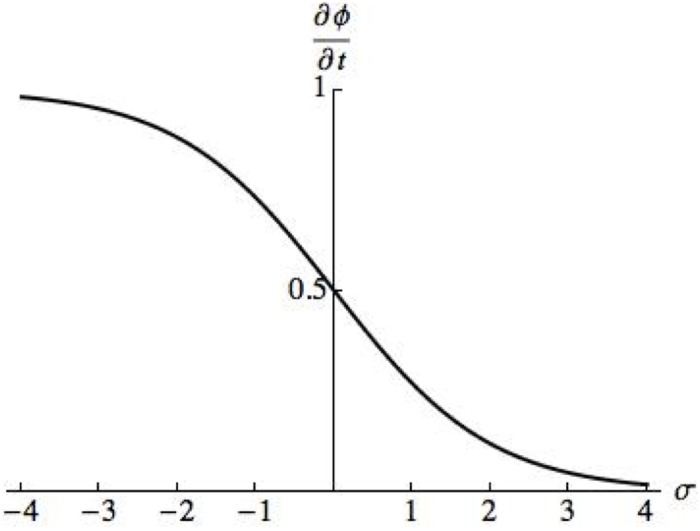
Graph of $\frac{\partial \phi }{\partial t}=C_{1}[\mu_{b}-\frac{\epsilon_{b}}{2}(\tanh(\frac{\beta }{2}\Delta G+\frac{\beta }{2}\sigma )-1)]$. In [15], specific parameter values are not provided and a dimensionless system of equations is solved. In the graph we use parameter values *C*_1_ = 1, *μ*_*b*_ = 0, *ϵ*_*b*_ = 1, *β* = 1, and Δ*G* = 0. Note that in the function graphed we add $\frac{\beta }{2}\sigma$, whereas in Eq (8) the term $\frac{\beta }{2}f_{a}$ is subtracted. This is because compressive stresses, which are assumed in [15] to activate FA formation, are negative, and *f*_*a*_ is denoted to be a positive force parameter.

The chemical potential of FA complexes in bulk is given by *μ*_*b*_. The second term on the right hand side describes the effects of the force applied to the FA by the cytoskeleton, denoted *f*_*a*_, in the chemical potential. The remaining terms on the right hand side describe how the chemical potential is affected by interaction between the FA complexes and by the addition of new FA complexes to the existing FAs. The energetic cost for a conformational change in the FA is given by Δ*G*, and the terms *C*_1_, *ϵ*_*b*_, *β*, *γ*(*f*_*a*_), *ϵ*, *c*, and *B* are parameters of the system. When one neglects the FA complex interaction terms and replaces the force *f*_*a*_ with stress *σ*, $\frac{\partial \phi }{\partial t}$ has the form that is graphed in Fig 2. This figure illustrates that Eq (8) captures the activation of FA complexes by compressive stresses (negative values of *σ*).

It has been established experimentally that increases in intracellular stresses arising from interaction with the substrate and stress fibers increase FA size [5, 13]. However, a specific functional dependence of FA growth rates on intracellular stress has not been established. Using Eq (8), Besser and Safran provide one mathematical description of how FA evolution is dependent on a constant force generated by actin stress fiber in a one-dimensional framework. We simplify their model by assuming that intracellular stresses affect the FA chemical potential and only compressive stresses affect FA activation while tensile stresses have no effect. This simplification allows us to extend the model for FA evolution to a more realistic two-dimensional description of the cell and to explicitly couple FA evolution to calculations of intracellular stresses. As a result, we are able to predict the stress-dependent growth of the FAs and, due to the attachment of the cell to the substrate via FA springs, account for the effect of the underlying substrate.

The equation governing the evolution of *ϕ* in our model is given by

$$\frac{\partial \phi (x,t)}{\partial t}=k_{\sigma }\tanh\left(-k_{2}\bar{\sigma }\left(x,t\right)\right)H(-\bar{\sigma }\left(x,t\right)$$ (9)

In Eq (9), evolution rate parameters are given by *k*_*σ*_ and *k*_2_, *H* is the Heaviside function. We define the average bulk stress by

$$\bar{\sigma }\left(x,t\right)=\frac{\sigma_{xx}\left(x,t\right)+\sigma_{yy}\left(x,t\right)}{2}$$ (10)

and note that this scalar value is also referred to as the hydrostatic component of the stress tensor, of which *σ*_*xx*_ and *σ*_*yy*_ are components. Eq (9) couples the intracellular stresses with the evolution of the FA. This evolution equation has a very similar structure to that illustrated in Fig 2 but results in a smaller number of parameters whose values must be determined. Due to the fact that the existing theoretical developments (e.g. [15]) offer little guidance concerning the parameters in Eq (9), and because there is no quantitative experimental data available which would allow us to formally determine values of *k*_*σ*_ and *k*_2_, we use a trial and error approach and obtain the following parameter values: *k*_*σ*_ = 360.0 min^−1^ and *k*_2_ = 1.0 kPa^−1^. These values of *k*_*σ*_ and *k*_2_ are chosen so that FA growth rates in response to intracellular stresses result in total sizes of adhesion area observable in live cells (see, for example, total FA areas in [13]). In addition, these value allow for tractable and efficient simulations. Such an approach is sufficient to demonstrate that the main assumptions of the model are viable. When quantitative experimental results become available, more formal approaches (e.g. regression methods) may be used to determine *k*_*σ*_ and *k*_2_.

FAs evolve according to Eq (9), which is coupled to the following rule

ϕ(x,t)≥0.5→FAspringisplacedatpositionx (11)

ϕ(x,t)<0.5→FAspringisremovedfrompositionx (12)

The total FA area is therefore calculated by determining the area over which *ϕ* ≥ 0.5.

In addition to modeling FA turnover according to Eqs (9), (11) and (12), we also incorporate a stretch-dependent FA rupture and a microtubule dependent rupture. In certain simulations, if the stretch in an FA spring surpasses a critical value, this attachment between the cell and the substrate breaks at this spring. In addition, FA evolution processes are also coordinated by the microtubule network [37, 38]. FA disassembly by microtubules is a very complex process that is not well understood. Possible mechanisms include microtubule-induced increase of cell contractility near an FA, transport of endocytosed integrins via microtubule motors, or microtubule transport of ECM degradation molecules (matrix metalloproteases) to the FA leading to the breakdown of the cell-ECM attachments [38]. Since it has been established that microtubules branch from the nuclear envelope to the cell periphery, we implement control of FA size into the model by assuming that these cytoskeletal structures disassemble FAs from the cell center out to the cell edge. We model the effects of FA disassembly due to microtubules by specifying a critical FA area to cell area ratio, and remove any FA springs that allow the FA area to exceed this critical ratio. Springs closest to the cell center are removed to keep the FA to cell area ratio below the critical level. The local value of *ϕ* is also lowered to 0.2 when a spring is removed, thereby modeling the disassembly of the entire focal adhesion complex.

### A note about numerical implementation

We solve the system of governing equations for the cell displacement, substrate displacement, cell stresses, and FA volume fraction (**u**_*c*_, **u**_*s*_, ***σ***_*c*_, and *ϕ*, respectively) using the finite element method for spatial discretization and, for the variables **u**_*c*_, **u**_*s*_, and ***σ***_*c*_, we use a backward difference scheme for time discretization. The evolution of the volume fraction of FA, *ϕ*, is discretized in time using an a forward difference method.

As biological cells move and spread they deform significantly, and some elements in the finite element mesh become significantly misshapen. This in turn leads to a lack of convergence of the Gauss-Seidel iterative solution approach used in the simulations. To overcome this issue, we incorporate remeshing in the numerical algorithm. Remeshing is performed either after a fixed number of time steps or when one of the triangular elements falls below a critical minimum height to area ratio. Since remeshing of the computational domain results in an additional numerical error, to be able to compare results of different simulations we force the remeshing to occur at fixed, predetermined time steps. In this manner, results that are being compared have been computed on finite element meshes that were remeshed the same number of times. Finally, we note that all programming was done in Matlab. Parameters used in simulations are listed in Table 1.

**Table 1:** Parameter values used in simulations.

Parameter	Description	Value(s)	Source
*E*_*c*_	Cell Young’s modulus	0.5–20 kPa	[39], [40]
*E*_*s*_	Substrate Young’s modulus	1.0–100 kPa	[39], [41], [40]
*ν*	Cell and substrate Poisson ratio	0.3	[42]
*k*_*a*_	FA spring constant	5 × 10^−5^ dynes/*μm*	[32]
*k*_*σ*_	FA evolution parameter	360 min^−1^	New Parameter
*k*_2_	Stress feedback of FA growth parameter	1.0 dynes^−1^	New Parameter

## Results

### Model validation

Here we validate our model by comparing the model predictions with experimental work. Traction force microscopy [43] allows the experimental determination of tractions exerted by a cell on a substrate during motility or spreading. We compare our numerical results to the traction patterns measured for a spreading human airway smooth muscle cell [39] and the traction patterns of a contracting cardiomyocyte [40]. The aim of these comparisons is to illustrate the fidelity of the model in its ability to correctly capture mechanical responses for two different cell types on substrates with different stiffnesses and chemical patterning resulting in different adhesion patterns and cell shapes.

As described in [39], smooth muscle cells were placed onto 50-*μ*m diameter circular islands made up of polyacrylamide gel with a collagen coating. Cells were plated on the islands and cultured for several hours, during which time they spread onto the island. We choose this particular experimental result as a model validation because the geometry of the cells spreading on the circular islands can be mimicked very closely in our numerical simulations. Smooth muscle cells are relatively soft with a Young’s modulus of approximately 0.1 to 0.5 kPa, and the polyacrylamide substrate used in [39] has a Young’s modulus of approximately 1.3 kPa. In the corresponding simulations we set the Young’s modulus to 0.5 kPa for the cell and 1 kPa for the substrate. Remaining parameter values for this and other simulations are listed in Table 1. After several hours of spreading, the cell diameter in the experimental results is approximately 40 *μ*m, so we compare the experimental results to our numerical simulations at 120 minutes.

**Fig 3:**
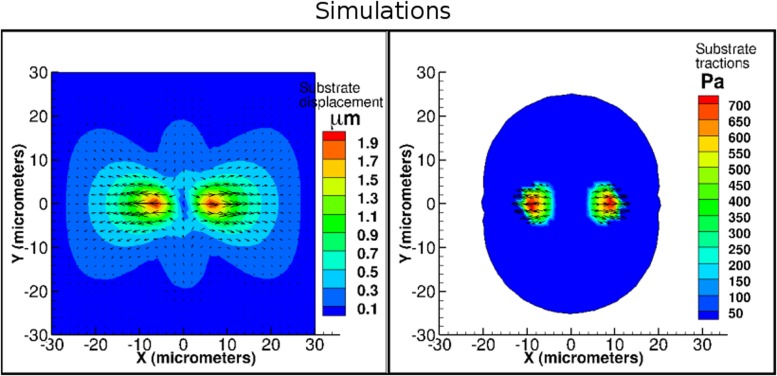
Comparison of simulations to cell spreading in a confined environment. Our simulations are shown and corresponding experimental results can be found in Fig 3 of Wang et al. [39] (see center and rightmost panel in middle row.) We illustrate numerically computed substrate displacements and FA tractions. FA traction magnitudes are shown as contours and traction vectors are also illustrated. We note that numerical results compare well to experiments both qualitatively and quantitatively. Cell and substrate Young’s modulus: *E*_*c*_ = 0.5 kPa, *E*_*s*_ = 1 kPa. FA springs do not rupture.

In Fig 3 we show numerical results for smooth muscle cell spreading. When these results are compared to experimental results shown in Fig 3 of Wang et al. [39], we observe maximum substrate displacements from both experiments and our simulations in the region where the cell is attached to the substrate. Quantitatively, the maximum experimentally determined displacements are approximately 3.5 *μ*m which are in good agreement with the maximum computed displacements of 2.0 *μ*m. Further, both experiments and the simulations show a a similar location of maximum in traction forces exerted by the cells onto the surface. The maximum experimentally determined tractions are approximately 500 Pa which are in reasonable agreement with the maximum computed tractions of 740 Pa.

The second experimental system that we pick to validate our model is a rat cardiomyocyte plated on a two dimensional silicone substrate on which they are reported to contract with an average frequency of 38 contractions per minute [40]. In this experimental work, the effect of a range of substrate stiffnesses varying between 1–500 kPa, specifically 1, 15, 30, 50, 150 and 500 kPa, is explored. Cardiomyocytes are known to exhibit a range of stiffnesses with the healthy cell having a Young’s modulus in the range of 10–30 kPa and, with aging and with different diseases of the heart, can go up to 150 kPa. The silicon substrate microstructure consists of a hexagonal lattice of microdots with an edge length of 2.5 *μ*m. For the purpose of analyzing their results the authors show that the cell shape can be approximated by a triangular region on the lattice. In line with this, we model the cell to have a triangular shape with an edge length of 30 *μ*m, which is comparable to the edge length reported in [40]. In simulations we compare the maximum traction at the peak of a single contraction. Here, we set the cell Young’s modulus to 20 kPa. Since results in [40] correspond to a wide range of substrate Young’s moduli, we ran simulations with four different substrate Young’s moduli (*E*_*s*_ = 15 kPa, 20 kPa, 25 kPa, and 50 kPa) and present results corresponding to a substrate Young’s modulus of 15 kPa since the predicted substrate displacement comparisons are the most accurate.

**Fig 4:**
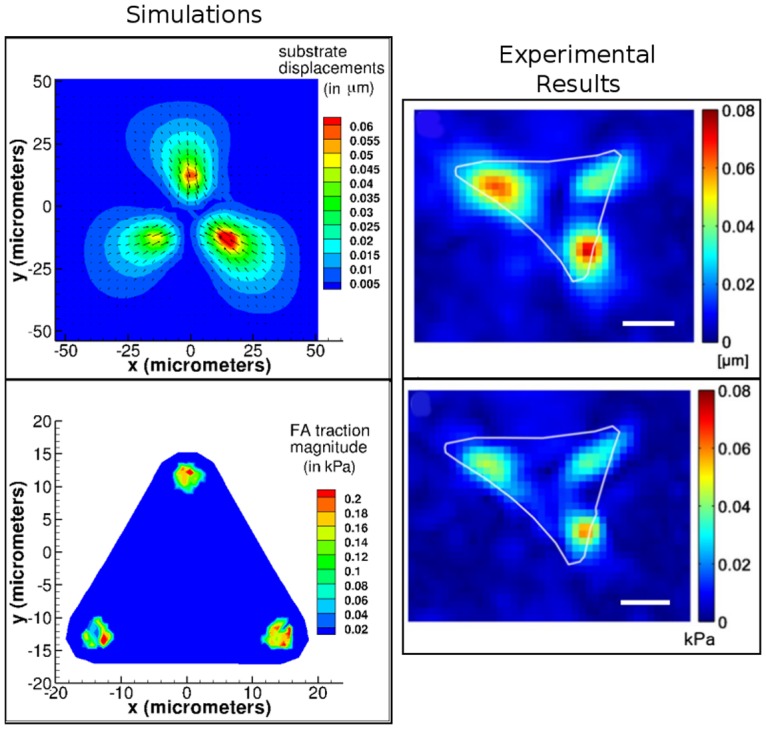
Comparison of simulations to contraction of a cardiomyocyte. Our simulations are shown in the left panel, and experiments from Hersch et al. [40] are on the right (*with permission under CC-BY 4.0 license*). Experimental result figures were modified from original by including scale bar and removing letter referring to original figure from top left corner. Scale bar = 10 *μm*. The substrate displacements from simulations compare well to those obtained experimentally both qualitatively and quantitatively. FA tractions, whose magnitudes are illustrated as contours, compare well qualitatively. Cell and substrate Young’s modulus: *E*_*c*_ = 20 kPa, *E*_*s*_ = 15 kPa. FA springs rupture at a stretch of 0.4 *μ*m.

As illustrated in Fig 4, computed maximum substrate displacements are 0.06 *μ*m and maximum experimentally measured substrate displacements are 0.08 *μ*m. The maximum FA traction magnitudes from the simulation are around 0.2 kPa while those from the experiments are around 0.08 kPa, i.e. approximately 2.5 times larger for numerically computed cardiomyocyte contraction than observed in experiments. This overestimation of the computed traction stress could be due to several factors, such as differences in the cell Young’s modulus or the fact that contractions in the experimental cell system are temporally periodic while in the simulation we run one steady contraction. The similar pattern of displacement and traction distribution indicates that the overall features of the model do reproduce experimental measurements.

The fact that the model predicts the substrate displacements accurately and captures the traction magnitudes reasonably well for two different cell systems on very different substrate stiffnesses gives us confidence that the model incorporates the essential physics that describe the dynamic mechanical interactions between an actively moving cell and a deformable substrate. In the next section we use this model of mechanical interactions between a spreading cell and substrate to generate some insights on why focal adhesions are typically observed on the cell periphery and not the cell center. Through this investigation we also seek to establish what are some behaviors of the cell this current model is able to capture and what are some shortcomings of the model in its current state. In the discussion section we propose some mechanisms that could potentially be added to the model to address some of these shortcomings.

### Effects of FA positioning on cell and substrate response

**Fig 5:**
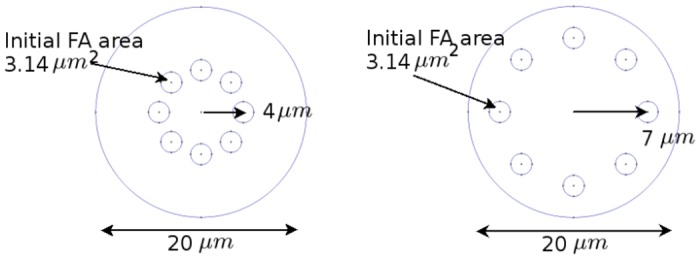
Initial FA configurations. Initial FAs are placed at a radial distance of *R* = 4 *μm* (left) or *R* = 7 *μm* (right). From this initial configuration FA location evolves in a stress dependent manner as described by Eq (9).

To gain a better understanding of why experimentally observed FAs are typically located on the cell’s periphery and not its center we construct two model systems. Both model systems begin with circular cells with radius of 10 *μ*m and eight FAs with an area of 3.14 *μ*m^2^ each. These FAs are placed either at a distance of *R* = 4 *μ*m or *R* = 7 *μ*m from the cell center, as illustrated in Fig 5. FAs originally placed at a distance of 7 *μ*m from the cell center are referred to as peripheral FAs and those that are at a distance of 4 *μ*m from the center are called central FAs. For these two model systems we investigate how global response of the cell and the substrate is affected by changes in FA size, FA strength, and substrate Young’s modulus. FA size is controlled by limiting the ratio of the total FA area to the cell area, as described in the methods section. FA strength is represented by either very strong binding to the substrate, where FA springs do not rupture, or weak binding, in which case FA springs break if they are stretched beyond 0.4 *μ*m. The global properties we investigate are substrate displacements, average bulk stresses within cells (as defined by Eq (10)), cell spread area, and total FA area. By comparing the variation of these global responses for the two model systems as a function of FA-cell area ratio, FA strength, and substrate stiffness, we show that there are certain potential advantages to having FAs at the periphery as opposed to the cell center.

**Fig 6:**
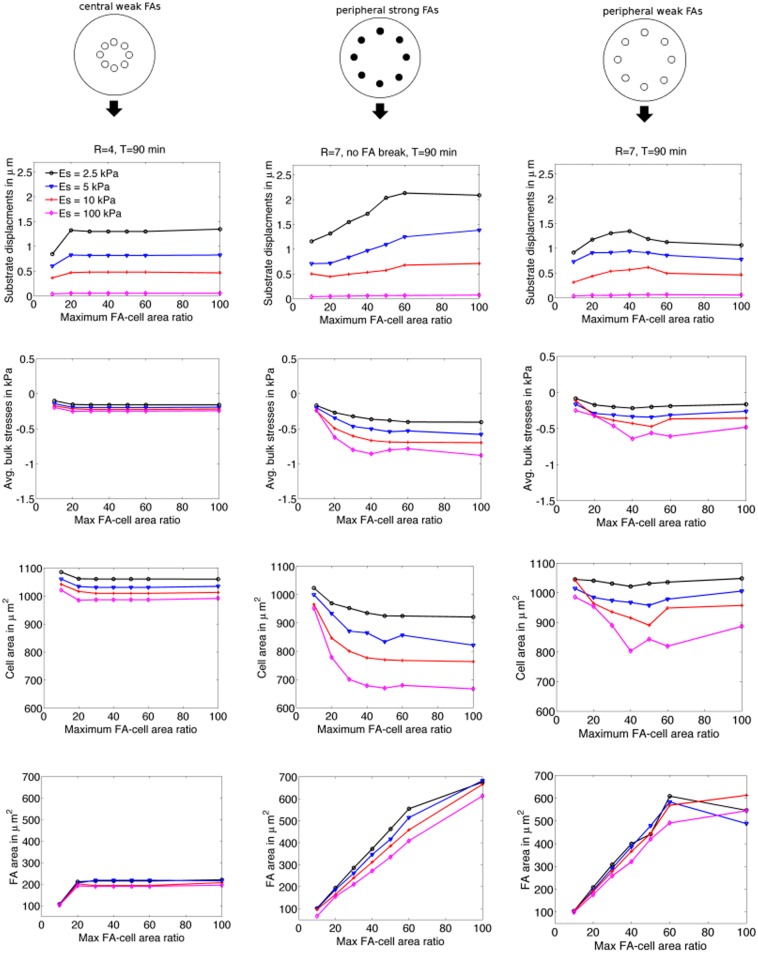
Effects of critical FA-cell area ratio and substrate stiffness on cell response. The horizontal axis represents variations in critical FA-cell area ratio, while line colors/markers correspond to different substrate stiffnesses; the legend for all figures is illustrated in the top left graph. The first column of graphs corresponds to data for cells with central, weak FAs (R = 4), the second column corresponds to peripheral, strong FAs (R = 7, no FA break), and the third column corresponds to peripheral, weak FAs (R = 7).

Fig 6 illustrates the effects of the FA-cell area ratio, FA strength, FA location, and substrate Young’s modulus on maximum substrate displacement (first row), average bulk stresses (second row), cell area (third row), and total FA area (fourth row). All quantities were computed after 90 minutes of spreading. Negative values of average bulk stresses correspond to compression and positive values correspond to tension. Each column corresponds to one of the three FA strength / FA position configurations we consider. In the first column we graph results for centrally located, weakly bound FAs. In the second column we consider peripheral, strongly bound FAs, and in the third column peripheral, weak FAs. The purpose of including these three cases is to determine which attribute, FA strength or position, modulates the global cellular and substrate responses more significantly.

We observe that substrate displacement decreases with an increase in substrate Young’s modulus (row one of Fig 6), which has been observed experimentally [44, 45] and is natural to expect. The sensitivity to substrate Young’s modulus is much larger for both weak and strong peripherally located FAs than it is for centrally located FAs. Substrate displacements are not as sensitive to the critical FA-cell area ratio as they are to substrate Young’s modulus, except in the case of strongly-bound, peripheral FAs. In cells with weakly bound FAs, maximum substrate displacements reach a maximum of approximately 1.5 *μ*m at a critical FA-cell area ratio of 20%-40%, and do not increase for larger FA-cell area ratios.

The second row of Fig 6 shows that average bulk stresses in cells with centrally located FAs are only slightly sensitive to substrate Young’s modulus and are effectively insensitive to changes in FA size beyond a FA-cell area ratio of 20%. The average bulk stresses within cells with peripherally located FAs are much more sensitive to changes in both substrate stiffness and increased the FA-cell area ratio. In this case, FA strength also plays an important role in modulating intracellular stresses. For strongly bound, peripheral FAs, the maximum average compressive stresses (i.e. most negative points on the graph) increases from 0.40 kPa to 0.88 kPa as the substrate Young’s modulus increases from 2.5 kPa to 100 kPa, while for weakly-bound peripheral FAs the same increase in substrate Young’s modulus leads to an increase in average compressive stresses from 0.21 kPa to 0.64 kPa. Similarly, as one increases the critical FA-cell area ratio for a cell spreading over a substrate with Young’s modulus of 100 kPa, for strongly bound FAs the average compressive stresses increase from 0.24 kPa to 0.88 kPa and for weakly bound FAs they increase from 0.25 kPa to 0.64 kPa. Modulating intracellular stresses is critical to a cell’s mechanosensing ability. These results indicate that cells can regulate intracellular stresses by three mechanisms: (i) by modulating the FA position, (ii) by modulating the substrate attachment strength, and (iii) by modulating FA size.

In rows three and four of Fig 6 we show how cell and total FA area is modulated by varying the substrate Young’s modulus and critical FA-cell area ratio. For centrally located FAs, the cell area decreases slightly with substrate Young’s modulus. However, the cell area is insensitive to changes in the FA-cell area ratio beyond 20%. We observe that the reason for the insensitivity beyond 20% is due to the fact that the FA area for cells with centrally located FAs does not grow above 19%-21% (depending on substrate Young’s modulus) of the cell area, even if the critical FA-cell area ratio is set to values above 20%.

In our model the cell spread area decreases with substrate stiffness, and this area decrease is more significant for cells with peripheral FAs. Many experimental results show that spread areas and FA sizes increase with substrate stiffness [46, 47]. However, given the nature of the current model, we expect the behavior that we observe, and we describe the mechanism for decreased cell spreading in the discussion section. For all substrate stiffnesses considered, the area of cells with peripheral FAs decreases with the FA-cell area ratio. This is due to the fact that a larger attachment area increases the net force acting on the cell by the attachments and confines the cell from spreading.

For both strongly and weakly bound peripheral FAs, the FA areas, which are determined by the locations at which the FA volume fraction, *ϕ*, is greater than 0.5, reach the maximum allowable sizes up to a critical FA-cell ratio of 60%. Therefore, the total FA size is approximately the same for both strongly and weakly bound cells. However, rows one through three of Fig 6 illustrate that substrate displacements and average bulk compressive stresses are smaller for weakly bound FAs than for strongly bound FAs. Therefore, these results suggest that the FA attachment strength is an important modulator of intracellular response.

**Fig 7:**
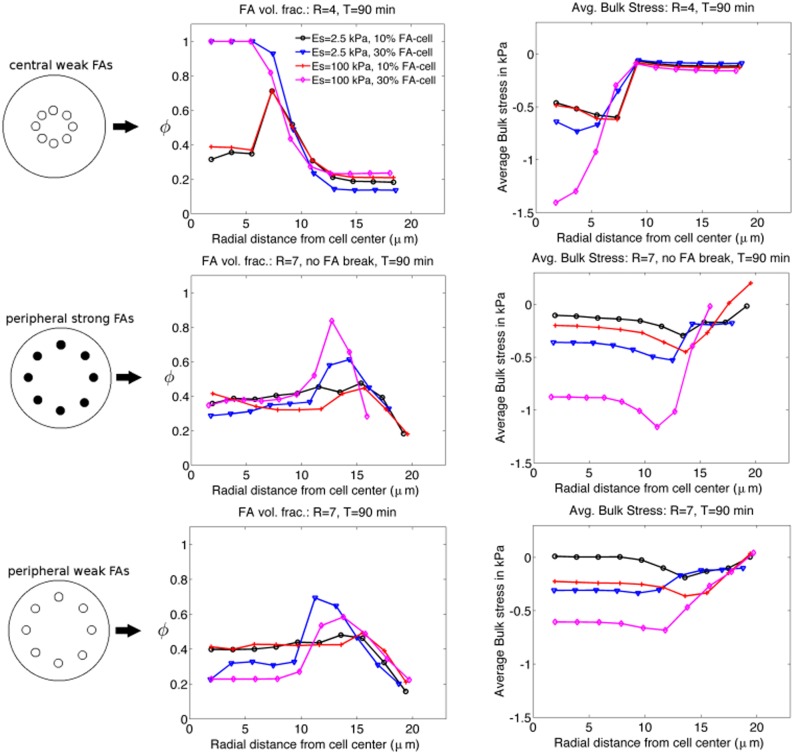
Dependence of FA volume fraction *ϕ* and bulk stress on spread cell radius. We consider four different substrate Young’s modulus, FA-cell area ratio combinations: black- *E*_*s*_ = 2.5 kPa, 10%, blue- *E*_*s*_ = 2.5 kPa, 30%, red- *E*_*s*_ = 100 kPa, 10%, magenta- *E*_*s*_ = 100 kPa, 30%. A legend for all graphs is in the top left panel, and the initial FA configurations for data illustrated in a given row are shown in the left-most column.

Fig 7 shows how the FA complex volume fraction and the average bulk stresses vary with the cell radius after 90 minutes of spreading. Values at each radial coordinate were computed by averaging *ϕ* and stress values in a ring of size Δ*r* centered at a given radial value, *r*. Row one of Fig 7 illustrates radial *ϕ* and stress values for central, weakly-bound FAs, row two for peripheral, strong FAs, and row three shows radial values for peripheraly located, weak FAs. We consider four FA size / substrate Young’s modulus combinations: substrate Young’s modulus (*E*_*s*_) of 2.5 kPa and critical FA-cell ratio of 10% in black circles, *E*_*s*_ = 2.5 kPa and FA-cell area ratio of 30% in blue triangles, *E*_*s*_ = 100 kPa and FA-cell area of 10% in red plus symbols, and *E*_*s*_ = 100 kPa and FA-cell area of 30% in magenta diamonds.

The values of *ϕ* (specifically, *ϕ* > 0.5) indicate that for peripheral FAs, the FAs remain localized in a ring near the periphery of the cell, while centrally located FAs grow to occupy the entire cell center and do not move towards the periphery. In general, compressive stresses are largest in cells with centrally located FAs. However, due to the central location of the FAs, significantly smaller portions of the cell are under a state of compression than in cells with peripherally located FAs. In other words, peripherally located FAs affect a larger portion of the cell than do centrally located FAs. Due to the fact that peripheral FAs have a larger zone of influence, the average compressive stresses in a cell with weak, peripheral FAs are larger than for cells with centrally located FAs (see row two in Fig 6).

**Fig 8:**
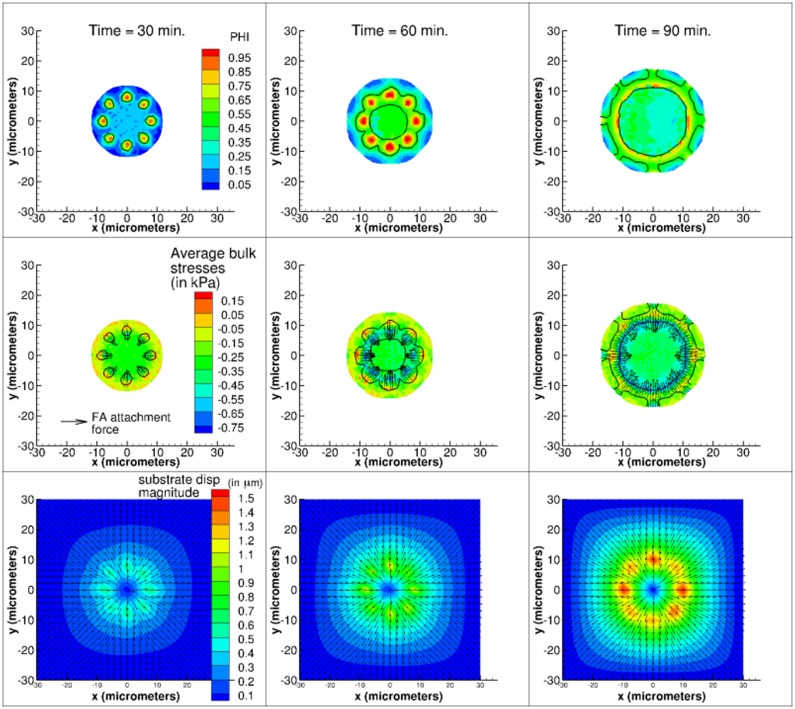
Spatiotemporal evolution of *ϕ* (top row), cell stress (middle row), and substrate displacement (bottom row) at time = 30, 60, 90 min. FA-cell area critical ratio: 30%; *E*_*s*_ = 2.5 kPa; No FA breakage. Black outlines indicate the location of the FAs. Vectors in cell stress contour graphs (middle row) show direction of forces arising from FA attachments. Note that as the cell spreads these forces are oriented toward the cell center.

To illustrate the ability of our model to capture temporal and spatial distributions of various intracellular and substrate variables, in Fig 8 we graph contour plots of the distribution of FA complex volume fraction, average bulk stresses, and substrate displacements at 30 minutes, 60 minutes, and 90 minutes of cell spreading on a substrate with Young’s modulus of 2.5 kPa. Here, we consider a cell with strongly-bound, peripheral FAs that are restricted to a FA-cell area ratio of 30%. This cell starts with eight distinct FAs that merge into a single FA ring that remains at the periphery of the cell, in contrast to cells with centrally-located FAs that merge into a single large circular FA in the cell center (see first row of Fig 7). Given the physics described in our model, as the cell spreads, the FA springs exert radially inward oriented forces on the cell, as shown by the vectors in the second row of Fig 8, and this leads to the formation of largest compressive stresses on the interior of the FA ring. Due to the fact that we assume that strong FAs do not break, substrate displacements increase with time.

## Discussion

In this paper, we describe a model of a spreading cell interacting mechanically with a two-dimensional substrate. The model of biochemical interactions is reduced to two basic components, an equation describing the evolution of an FA complex volume fraction, *ϕ*, and a prescription of an active rate of deformation tensor, **D**^*A*^, which describes cell spreading. In this version of the model, we assume that the spreading rate is uniform and constant, i.e. it does not directly depend on the intracellular stress profile or on variation in concentrations of any cytoskeletal components. The model allows for such a dependence to be introduced, and developing a spatially varying active rate of deformation tensor is part of our future work.

Using this model, we can capture spatial and temporal variation in various substrate and cellular fields, such as substrate displacement, FA tractions, FA volume fraction, and intracellular stresses. In future work, this capability will allow us to predict cellular response to inputs that vary spatially or temporally, including spatially inhomogeneous chemical patterns on functionalized surfaces or cyclic strains applied to the cell. Furthermore, this simplified model captures experimentally observed substrate displacement and cell tractions patterns, as seen in Figs 3 and 4, and allows us to elucidate some fundamental features related to FA positioning.

### Why does our model predict a decrease in cell spread area with increasing substrate stiffness?

Given the physical interactions described in our current model, we find that as one increases substrate stiffness, FA size, or FA attachment strength, the cell spread area decreases. The mechanism for decreased cell area with increased substrate Young’s modulus is as follows. When a cell spreads over a stiffer substrate, the stretch in the FA springs increases due to the lack of compliance in the substrate, which is illustrated in row one of Fig 6 where we can see decreased substrate displacements with an increase in substrate Young’s modulus. The increase in FA spring stretch results in mechanical force oriented toward the cell center which resists spreading, and this in turn leads to a smaller spread area over a stiffer substrate.

### Substrate displacement and cellular bulk stress increase with FA size and strength

For a given substrate Young’s modulus, an increase in FA size results in an increase of the total cell area over which deformations are constrained. This constraint is associated with an increase in the total force acting on the substrate and the cell. Hence, FA size increases lead to an increase in substrate displacement and intracellular bulk stresses. This is consistent with experimental observations in Rape et al. [13], in which it is shown that FA size increases are commensurate with an increase in intracellular stresses. Simultaneously, the increase in FA size and the accompanying increase in total force acting on the cell causes a decrease in cell spread area because the mechanical resistance to spreading increases.

Not surprisingly, we also see that increasing FA strength results in increases in substrate displacement and intracellular stress. Again, stronger FAs allow for an increased FA spring stretch and larger forces from the FAs acting on the cell. In the case of weak FAs, FA springs break at a critical stretch partially releasing constraints imposed on the cell. However, since FA volume fraction levels are sufficiently high, even after an FA spring ruptures, ruptured FA springs immediately reattach (with zero stretch). Effectively, weak FAs break and reform on the periphery of the cell. On average, the force from these weak FAs is smaller than the force from strong FAs, and this leads to lower substrate displacements, lower intracellular stresses, and a lower mechanical resistance leading to larger cell spread areas.

### An explanation of why peripheral FAs result in larger stresses than central FAs

Elineni and coworkers highlight that the location of FAs, i.e. peripheral vs. dispersed throughout the cell, is critical to adhesion strength and mechanotransduction [10]. Our model and simulations show that as the location of FAs shift from center to periphery, the cell’s sensitivity to changes in the substrate Young’s modulus and FA size markedly increases. When FAs are at the center of the cell, substrate displacements, average bulk stresses, and cell spread areas plateau at a FA-cell area ratio of 20%, beyond which an increase in the critical FA-cell area ratio does not affect the substrate and cell response. To explain this behavior we examine the radial variation of the average bulk stresses and the FA volume fractions in Fig 7. For the central FA case the maximum compressive stresses are at the cell center and confined to the region inside the ring of initial FAs, which can be compared to central (6 *μ*m) solid FAs in Elineni et al. These FAs do not spread to the periphery and grow only into the cell center restricting the size the total FA area can reach. When FAs are at the periphery the maximum compressive stresses are localized at the periphery, as shown in Fig 7, and though the magnitude of maximum compressive stresses are lower than those observed for central FAs, the entire region interior to the FAs remains in a state of compression. In addition, as seen in Fig 8, FAs that start at the periphery remain in the periphery as the cell spreads.

**Fig 9:**
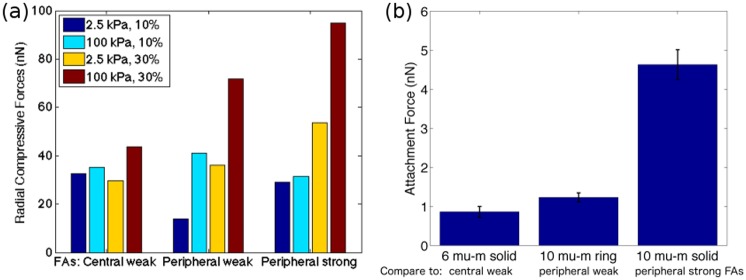
Comparison of computed total compressive forces and experimentally measured attachment forces. (a) Radial compressive forces are determined from the computed radial stress data illustrated in Fig 7. Radial compressive forces are obtained by integrating the average bulk stresses over the cell area and averaging over the angle 2*π*. Different colors correspond to different combination of substrate Young’s modulus and critical FA-cell area ratio. (b) Attachment forces computed from adhesion strength data in Fig 5 in [10]. Attachment forces were computed by multiplying experimentally measured attachment strengths by total attachment area. Attachment strengths were determined by counting remaining numbers of adherent cells at the end of a spinning platform experiment. Three attachment configurations were considered: A 6 *μm* circular patch with a total adhesive area of 28 *μm*^2^, a 10 *μm* ring with a total adhesive area of 28 *μm*^2^, and a 10 *μm* circle with total adhesive area of 78 *μm*^2^. Each spinning platform experiment was performed in triplicate. Attachment forces computed from average attachment strengths and corresponding standard deviations are illustrated. More details can be found in [10]. Experiments confirm that as total attachment area increases and as attachments form closer to the periphery, attachment forces increase.

These results together suggest that the presence of FAs at the periphery results in a greater zone of influence within the cell. The radial compressive forces illustrated in Fig 9a are a measure of the total force acting on the cell, and the total forces within cells with peripheral FAs vary significantly more when comparing different FA sizes and substrate Young moduli than total forces in cells with central FAs. Ultimately, a larger zone of influence manifests itself as the enhanced sensitivity of both cellular and substrate responses. In Fig 9 we see that larger inward radial forces in cells with peripheral FAs than with cells with central FAs are in line with experiments. The experimental results of Elineni et al. [10], which are shown in Fig 9b, provide a measure of the total adhesion forces which are shown to increase with distance from the center and size of FAs. The larger radial force in our study results from the increase in the force from the FA springs. Since FA force is directly related to FA adhesion strength, these two quantities in essence provide us with similar information. Further the experimental results of Elineni et al. corresponding to the two solid patches (6 *μ*m and 10 *μ*m) correspond qualitatively to the cases of larger FA sizes (e.g. 10% and 30%), and our results show that cells with larger FAs result in larger radial forces. The actual magnitudes of the forces are different since the experiments provide information about the interfacial forces while from the simulations we report the forces acting within the cell. *Given the enhanced sensitivity of cellular responses when FAs are located at the cell periphery we speculate that cells evolved to take advantage of this aspect to have better mechanochemical control to the environmental changes by limiting FAs to the periphery*.

### Addressing shortcomings in the current model

It has been shown in experiments that for several cell types cell spread areas increase with increased substrate Young’s modulus. Yeung et al. showed that the circumference and area of NIH3T3 fibroblasts and bovine aortic endothelial cells increases with substrate shear storage modulus, while the circumference of human blood neutrophils stays essentially constant [46]. Tee et al. illustrate similar increases in spread area in human mesenchymal stem cell with increasing polyacrylamide gel rigidities [47]. These results suggest that our existing model must be augmented with a more detailed description of the cell’s cytoskeleton. Specifically, the cell spreading rate will likely need to include a description of the growth of a branched actin network, that is possibly dependent on intracellular tensile stresses, outside the FA region at the cell periphery. The addition of this machinery to our model will allow for compressive stresses at the FAs to strengthen the attachment of the actin filament to the FAs while at the edge of the cell the actin filament should undergo an enhanced rate of polymerization leading to a larger spread area. The formation of stress fibers is also strongly correlated with increased cell spread area [47, 48]. Stress fibers contract in a stress-dependent manner, and their contraction coupled with substrate compliance leads to smaller spread areas on softer substrates. The addition of such effects into the model will also result in cell areas more in line with those observed in experiments.

The modifications described above are part of the next set of changes that are currently being planned to the model. Further, the current model deliberately omits the details of the biochemistry behind the FA growth and the signaling resulting in the activation of the actin filaments. Our expanded model will include some of these details to capture the behavior of cells more realistically. In addition, the expanded model will allow us to investigate how modifying the biochemical network affects cell spreading.

## Conclusions

A key insight from the model described in this work is that mechanically a cell can take advantage of at least three levers that can modify its state of intracellular stress. These three levers are i) the FA size, ii) the FA attachment strength, and iii) the FA position. We have clearly shown that the FA position is critical to a cell’s mechanosensitivity, and cells exhibit far more sensitivity to the extracellular environment, the substrate Young’s modulus, when the FAs are found at the cell periphery. Our results may form a mechanical justification of what is observed in Chen et al. [9], Elineni et al. [10], and Prager-Khoutorsky et al. [11], in that FAs are often found in the cell’s periphery because this FA location makes the cell more sensitive to its microenvironment.

While we have noted various extensions of this model that we are planning for future work, the power of the framework as it exists is evident in that we have a two-dimensional description of the cell-FA-substrate system with the ability to model the interaction of different cell types with substrates of varying stiffness as demonstrated in the model validation section. The FAs in the model are dynamic in that they can grow and decay, and we are able to control their size and attachment strength. Furthermore, the model captures spatial distributions of substrate displacements and stresses, cellular deformations, and intracellular stresses, allowing us to predict these fields with reasonable accuracy.
